# Reduced Amygdala Volume Is Associated with Deficits in Inhibitory Control: A Voxel- and Surface-Based Morphometric Analysis of Comorbid PTSD/Mild TBI

**DOI:** 10.1155/2014/691505

**Published:** 2014-03-03

**Authors:** B. E. Depue, J. H. Olson-Madden, H. R. Smolker, M. Rajamani, L. A. Brenner, M. T. Banich

**Affiliations:** ^1^Institute of Cognitive Science, University of Colorado at Boulder, Boulder, CO 80309, USA; ^2^Department of Psychology and Neuroscience, University of Colorado at Boulder, Boulder, CO 80309, USA; ^3^VISN 19 Mental Illness Research Education and Clinical Center, Denver, CO 80220, USA; ^4^University of Colorado Denver, Anschutz Medical Campus, Aurora, CO 80045, USA

## Abstract

A significant portion of previously deployed combat Veterans from Operation Enduring Freedom and Operation Iraqi Freedom/Operation New Dawn (OEF/OIF/OND) are affected by comorbid posttraumatic stress disorder (PTSD) and mild traumatic brain injury (mTBI). Despite this fact, neuroimaging studies investigating the neural correlates of cognitive dysfunction within this population are almost nonexistent, with the exception of research examining the neural correlates of diagnostic PTSD or TBI. The current study used both voxel-based and surface-based morphometry to determine whether comorbid PTSD/mTBI is characterized by altered brain structure in the same regions as observed in singular diagnostic PTSD or TBI. Furthermore, we assessed whether alterations in brain structures in these regions were associated with behavioral measures related to inhibitory control, as assessed by the Go/No-go task, self-reports of impulsivity, and/or PTSD or mTBI symptoms. Results indicate volumetric reductions in the bilateral anterior amygdala in our comorbid PTSD/mTBI sample as compared to a control sample of OEF/OIF Veterans with no history of mTBI and/or PTSD. Moreover, increased volume reduction in the amygdala predicted poorer inhibitory control as measured by performance on the Go/No-go task, increased self-reported impulsivity, and greater symptoms associated with PTSD. These findings suggest that alterations in brain anatomy in OEF/OIF/OND Veterans with comorbid PTSD/mTBI are associated with both cognitive deficits and trauma symptoms related to PTSD.

## 1. Introduction

Posttraumatic stress disorder (PTSD) affects a significant percentage (e.g., 10–30%) of deployed combat Veterans (i.e., Operation Enduring Freedom and Operation Iraqi Freedom/Operation New Dawn (OEF/OIF/OND); [1, 2]). Similarly, a significant percent (e.g., 15–25%) of OEF/OIF Veterans are also affected by traumatic brain injury (TBI; [3, 4]), particularly mild TBI (mTBI). Therefore, estimates of comorbidity are as high as 42% [3–5]. One factor cited for the increase in the rise of comorbidity is the presence of implemented explosive devices (IEDs; [6]). Neuroimaging research indicates that individuals with PTSD exhibit abnormalities in the hippocampal/amygdalar complex [7, 8] and regions putatively responsible for regulation of them. That is the ventromedial prefrontal cortex (vmPFC) and the subgenual anterior cingulate cortex (sgACC) [8, 9]. Likewise, individuals with TBI that experience direct impact or blast wave trauma exhibit damage to the brain in these same regions, most significantly the vmPFC/sgACC [10–12]. The localization of injury associated with TBI may result from inner-cranial wave physics [13] and the presence of boney protuberances on the inner surface of the skull near the orbital and anterior temporal lobes [14, 15] makes the vmPFC/sgACC and amygdalar complex vulnerable. Therefore, it is not surprising that a vast anatomical and functional neuroimaging literature exists that focuses on or indicates results relating either PTSD or TBI to these brain regions. However, in comparison, there is a paucity of literature examining the neuroanatomical deficits in comorbid PTSD/TBI.

Neuroimaging studies examining comorbid PTSD/TBI are almost nonexistent, perhaps, because of an old but common notion that an amnesic TBI event was “protective” towards developing PTSD [16]. This idea has been largely reversed in the last 20 years by studies investigating the prevalence of cooccurring PTSD/TBI, which indicate increased rates of PTSD among individuals with a TBI when compared to individuals who have never had a brain injury [17, 18]. Furthermore, recent indications suggest that an occurrence of TBI may even render individuals more susceptible to PTSD [6, 17]. The few neuroimaging studies investigating comorbid PTSD/TBI indicate glucose hypometabolism in the cerebellum and medial temporal lobe [19] and increases in hemosiderin deposits (iron deposits related to hemorrhaging) linked to increases in TBI symptoms [20].

However, neuroimaging studies conducted on individuals with PTSD consistently indicate both functional and anatomical alterations in the vmPFC/sgACC, striatum, thalamus, and amygdalar/hippocampal complex (for reviews/meta-analyses see [21–24]). Functional studies show alterations in a multitude of brain regions, although being able to detect altered engagement likely depends on the tasks employed. As such, there are inconsistencies in reported results with both hyper- and hypoactivation observed in the aforementioned regions [25, 26]. However, anatomical studies consistently indicate reductions in amygdalar/hippocampal complex and the vmPFC/sgACC volume and represent the most replicated findings among individuals with PTSD, when compared to controls ([27–31].) Dysfunction in the vmPFC/sgACC—amygdalar/hippocampal complex pathways is suggested to be functionally associated with decreased control or regulation over fear/threat related stimuli and conditioning [25].

Compared to PTSD, the neuroimaging literature is less abundant concerning TBI, particularly mild TBI, and studies tend to focus more on anatomical measures (e.g., diffusion tensor imaging (DTI), morphometry) rather than function, possibly due to the condition acutely causing tissue damage [32]. Mirroring morphometric studies in PTSD, volumetric reductions in the amygdalar/hippocampal complex, and the vmPFC/sgACC occur in individuals with TBI as compared to controls [33–36]. Historically, TBI has been linked to impaired executive function, such that individuals with a TBI perform worse on tasks designed to tap executive or cognitive control processes (thought to primarily involve the PFC) than individuals without TBI [37, 38]. However, many of these studies involve individuals with moderate and severe TBI, rather than mTBI [39]. Therefore, it is unclear whether persistent executive dysfunction is also compromised among those with mTBI [5, 40].

Regardless of the paucity of neuroimaging studies examining individuals with comorbid PTSD/TBI studies, a reasonable estimate is that disruption in neural circuitry of comorbid PTSD/TBI likely involves constituent elements observed within singular diagnosis of PTSD and TBI individually. The most consistent and replicated findings common to both of these populations are anatomical alterations in the vmPFC/sgACC and amygdalar/hippocampal complex. Consistent with these anatomical findings, diagnostically comorbid PTSD/TBI is associated with deficient inhibitory control [41, 42]. Inhibitory control is ubiquitously described under the rubric of executive function as the ability to “hold back” or inhibit a prepotent response and its dysfunction is usually associated with impulsive symptomatology [43]. While anatomical findings indicate that regions of the brain underlying inhibitory control are compromised in both PTSD and TBI, it is unclear whether symptoms related to PTSD or TBI are more indicative of these inhibitory control deficits in comorbid PTSD/TBI.

While specific symptoms and diagnostic criteria associated with PTSD and TBI have been associated with amygdalar/hippocampal complex hyperactivity and reduced volume [22, 25, 44, 45]) as well as vmPFC/sgACC hypoarousal and decreased volume [25, 31, 46], however, there have been few anatomical neuroimaging investigations of the basic underlying cognitive processes that may be disrupted, without using emotional or fear-related stimuli. Knowledge of these more general cognitive processes and the associated alterations in anatomy is important in order to design more effective interventions. For example, it is thought that alterations in the putative fear conditioning/extinction circuitry (vmPFC/sgACC-amygdala) reflect poor inhibitory control. However, investigations, using tasks designed to tap inhibitory control by a means not associated with fear and/or threat, are lacking. One notable exception is a study by Falconer and colleagues [47] who investigated inhibitory control in individuals diagnosed with PTSD using a Go/No-go paradigm. Compared to controls, individuals with PTSD had decreased right inferior PFC activity and increased striatal activity, suggesting decreased activity in the neural mechanisms of inhibitory control. More research investigating inhibitory control as a cognitive or behavioral construct is needed to determine whether inhibitory systems are specifically linked to fear or threat related stimuli are altered in individuals with PTSD or whether they are generally compromised. Additionally, because deficits in inhibitory control are related to both PTSD and TBI, understanding the specific context in which inhibitory control is deficient may lend insight into dysfunctional neural mechanisms that are associated with the specific diagnoses.

In order to increase our understanding of the neural underpinnings of inhibitory control deficits in comorbid PTSD/mTBI, we investigated several questions. First, we investigated whether anatomical differences in previously deployed OEF/OIF/OND combat Veterans with comorbid PTSD/mTBI were consistent with either singular diagnostic (a) PTSD and/or (b) mTBI or they were perhaps more severe due to comorbidity, as compared to previously deployed OEF/OIF combat Veterans without PTSD or mTBI diagnoses. Because the extant literature covering both diagnostic groups indicates disruptions in general fear circuitry, we expected to find decreased anatomical volume within (a) vmPFC/sgACC and the (b) hippocampus/amygdalar complex. Second, we examined the degree to which performance on a task of inhibitory control that does not involve fearful or threatening stimuli, the Go/No-go task, was predicted by anatomical alterations. Finally, we examined the degree to which self-report of impulsivity and symptoms related to PTSD or mTBI are associated with anatomical alterations. Based on the extant literature, we hypothesized that individuals with PTSD/mTBI would show reduced volume in vmPFC/sgACC and amygdalar/hippocampal regions as compared to controls. Moreover, we predicted that anatomical alterations would predict behavioral measures. More specifically, we expected that decreased volume in these regions in individuals with comorbid PTSD/mTBI, but not controls, would be associated with decreased inhibitory control on the Go/No-go task, and with self-reported measures of impulsivity.

## 2. Methods

Twenty-one previously deployed OEF/OIF/OND combat Veterans with comorbid PTSD/mTBI diagnoses (20 males) and 17 OEF/OIF/OND previously deployed combat veteran controls without PTSD or mTBI (14 males) took part in the study. One individual (male) from the control group was excluded for MRI head movement leaving an N of 21 and 16, respectively. Recruitment was primarily accomplished through fliers circulated in the Denver area. All individuals were required to provide consent, which was approved through the Colorado Multiple Institutional Review Board. Individuals were compensated for their participation. Demographic information regarding age, gender, race, and years of education was obtained.

### 2.1. Recruitment

*Inclusion Criteria.* Inclusion criteria include (1) population between the ages of 18–45, (2) at least one OEF/OIF/OND deployment, and (3) population currently receiving or eligible to receive physical and/or mental health care through the VA Eastern Colorado Health Care System.

*Exclusion Criteria.* Exclusion criteria include (1) history of other significant neurological diseases (other than mild TBI for the appropriate group) as assessed by interview and chart review; (2) history or diagnosis of lifetime moderate or severe TBI for the PTSD/mTBI group, or any history of TBI for the non-TBI group, as assessed by interview and chart review; (3) history or diagnosis of nonactive duty-related mild TBI or PTSD disorder as assessed by interview and/or chart review; (4) diagnosis of schizophrenia or bipolar I disorder as assessed by administration of the Structured Clinical Interview for the DSM IV (SCID; [48]); (5) Computerized Assessment of Response Bias (CARB) [49] performance categorized as very poor effort, poor effort, or symptom exaggerator; (6) problematic drinking behavior that consistently exceeds recommended drinking limits per day, for example, diagnosis of alcohol abuse disorder or alcohol dependence disorder per the SCID, or five or more alcoholic drinks per day, four out of seven days per week for the previous two weeks; (7) use of illicit substance(s) more than five times in the two weeks before enrollment; (8) inability to read the informed consent document or adequately respond to questions regarding the informed consent procedure; (9) contraindication to having an MRI; and (10) Veterans who have previously enrolled in other VA studies which administer identical or similar instruments to this study.

*Diagnostic Criteria/Measures.* We used the Computerized Assessment of Response Bias (CARB) to assess performance and determine effort, the Shipley 2 Institute of Living Scale to measure premorbid level of functioning [50]. To assess impulsivity we used the Barratt Impulsivity Scale [51]. Alcohol and Substance use was measured by the SCID [48]. To diagnose PTSD we used the SCID and we also assessed PTSD symptom severity using the Trauma Symptom Inventory (TSI; [52]). To diagnose TBI we used the Ohio State University Traumatic Brain Injury Identification Method (OSU TBI-ID) structured clinical interview, which allows for interrogation of mTBI symptoms [53]; if the participant had a TBI, it must have been a mild TBI from active duty. Though severity of TBI by the OSU TBI-ID is mostly determined according to loss or alteration of conciousness, the following criteria was used to determine TBI severity: (1) mild TBI: A TBI with normal structural imaging, 0–30 minutes of loss of consciousness (LOC), a moment and up to 24 hours of alteration of consciousness/mental state (AOC), 0-1 day of or posttraumatic amnesia (PTA), or a best available Glascow Coma Scale Score (GCS) of 13–15 recorded within the 24 hours of the injury event, (2) moderate TBI: A TBI with normal or abnormal structural imaging, >30 min and <24 hours of LOC, >24 hours of AOC, >1 and <7 days of PTA, or a GCS score of 9–12, and (3) severe TBI: A TBI with normal or abnormal structural imaging, >24 hours of LOC, >24 hours of AOC, >7 days of PTA, or a GCS score < 9.

*Inhibitory Control.* To assess inhibitory control we used a standard Go/No-go task [54]. Participants were required to press the response button with the right index finger for each letter that appeared on the screen except for the letter X. The task consisted of three blocks of 120 trials each. Each letter, approximately 2.5 cm in size, appeared for 500 milliseconds with an interstimulus interval of 2000 ms. The letter “X” occurred on 20% of all trials (*n* = 72), which were presented randomly throughout the run. Other letters were randomly selected from the alphabet. Performance measures on the Go/No-go task were mean reaction time for correct go trials, errors of omission, and errors of commission.

### 2.2. Neuroimaging Acquisition

*Structural.* All structural MRI images were acquired using a Philips 1.5-Tesla Achieva 16-channel MR scanner located at the Denver Veterans Affairs Medical Center. An eight-channel headcoil was used for radiofrequency transmission and reception. Foam padding was placed around the head, within the head coil, to limit head motion during the scan. Structural images were obtained via a T1-weighted 3D TFE in 160 sagittal slices. Imaging parameters were as follows: echo time (*T*_*E*_) = 3.2 ms., repetition time (*T*_*R*_) = 7100 ms, flip angle = 8.0°, field of view (FoV) = 240 mm, and voxel size = 1.0 × 1.03 × 1.0 mm. Scan parameters were consistent for all imaging sessions.

### 2.3. Neuroimaging Analysis

*Voxel-Based Morphometry (VBM).* All VBM analyses were performed using the FSL-VBM toolbox and follow the processing pipeline put forth by Ashburner and Friston [55] and Good et al. [56]. First, the raw T1-weighted images were brain-extracted using the FSL default BET brain extraction process, which strips the skull and removes any nonbrain tissue from the image using the FAST4 tool. The resulting GM images were then aligned to MNI152 standard space using the affine registration tool FLIRT, followed by nonlinear registration using FNIRT. The resulting images were averaged to create a study-specific template, to which the native GM images were then nonlinearly reregistered using FNIRT. The registered partial volume images were then modulated (to correct for local expansion and contraction) by dividing the Jacobian of the warp field. The modulated segmented images were then smoothed with an isotropic Gaussian kernel with a sigma of 2, yielding full-width half-maximum (FWHM) 2 × 2.3 mm = 4.6 mm FWHM. The resulting subject-specific GM probability maps were input into a general linear model (GLM) evaluating group differences between all voxels of GM, using whole-brain GM volume as a nuisance covariate. One-sample *t*-tests for group contrasts were performed using the Threshold-Free Cluster Enhancement (TFCE) method, which detects clusters of contiguous voxels without first setting an arbitrary statistical cut-off (e.g., *Z* > 2.58) and controls the familywise error (FWE) rate at *P* < 0.05. Each contrast underwent 5000 permutations. Randomize produces corrected 1-*p* maps, which we used to mask *t*-score maps for all figures. Figures of statistical maps were created using FSLview. 

*Surface-Based Morphometry (SBM).* Automated segmentation of the bilateral amygdala and hippocampus was performed using FIRST (FSL v4.0.1) which uses a Bayesian probabilistic approach. The shape and appearance models in FIRST are constructed from a library of manually segmented images. The manually generated labels are parameterized as surface meshes and then modeled as a point distribution. Using the learned models, FIRST searches through shape deformations that are linear combinations of the modes of variation to find the most probable shape instance given the observed intensities from the input image. Using T1 images, the segmentation was performed with two-stage affine transformation to standard space of MNI 152 at 1 mm resolution [57, 58]. The first stage utilized a standard 12 degrees of freedom registration to the template and the second stage applied 12 degrees of freedom registration using an MNI152 subcortical mask to exclude voxels outside the subcortical regions. Boundary voxels were thresholded at *s* = 2 and *s* = 3, along with the recommended number of modes (iterations) for the hippocampus (30) and amygdala (50). All processes of segmentation were then visually inspected to assess boundaries by two independent raters for each of the two boundary thresholded training sets (*s* = 2,   *s* = 3). Because *s* = 2 yielded a more conservative boundary threshold that included the amygdala and hippocampus proper, while minimizing neocortical tissue, ventricles, and white matter, this data set was selected for final analyses. One-sample *t*-tests for group contrasts were performed using the Threshold-Free Cluster Enhancement (TFCE) method, which detects clusters of contiguous voxels without first setting an arbitrary statistical cut-off (e.g., *Z* > 2.58) and controls the familywise error (FWE) rate at *P* < 0.05. Each contrast underwent 5000 permutations. Randomize produces corrected 1-*p* maps, which we used to mask *t*-score maps for all figures. Figures of statistical maps were created using FSLview.

*Multiple Regression.* To perform multiple regression we used a two-stage procedure as outlined in Hastie et al. [59]. We first used penalized regression using LASSO [60] to perform subset variable/feature selection. Subsequently, because LASSO can over penalize highly collinear variables, we then performed an ordinary least squares (OLS) best model multiple regression on the subset of selected variables/features taken from LASSO to obtain beta estimates, regression coefficients, and determinants of explained variance.

## 3. Results

### 3.1. Demographic and Behavioral Measures

There were no significant differences between the groups in demographic characteristics (Table 1). Significant group differences emerged for previous alcohol use (*P* < 0.005), indicating that the PTSD/mTBI group had higher proportions of alcohol use. Significant group differences emerged for the Barratt Impulsivity Scale (Barratt) in the subcomponents of attention, perseveration, and self-control (*P* = 0.0002, *P* = 0.01, and *P* = 0.001, resp.), indicating that the PTSD/mTBI group exhibited higher proportions or more impulsivity than the control group. Group differences also arose in the Trauma Symptom Inventory in the three subcomponents linked to SCID PTSD diagnostic criteria (Intrusive Experiences, Defensive Avoidance, and Dissociation; all three *P* < 0.0001), indicating that the PTSD/mTBI group exhibited higher proportions or more symptoms associated with PTSD than the control group. Group differences were also found in the Shipley 2 Abstraction and Composite A score (*P* = 0.004, *P* = 0.006, resp.), indicating that the PTSD/mTBI group exhibited lower premorbid functioning as compared to the control group.

### 3.2. Inhibitory Control

Go/No-go behavior indicated a significant group difference in errors of commission (*t* ^2,35^ = 2.61, *P* = 0.009), with individuals with PTSD/mTBI (*M* = 15.14, SE = 1.82) who made more errors of commission than controls (*M* = 9.25, SE = 1.25). Errors of omission (EO) and reaction time (RT) did not significantly differ between the two groups (PTSD/mTBI, EO = 5.3, RT = 373.62 ms.; controls, EO = 4.4, RT = 409.77 ms., *P* = 0.78, *P* = 0.21, resp.).

### 3.3. Neuroimaging

**Figure 1:**
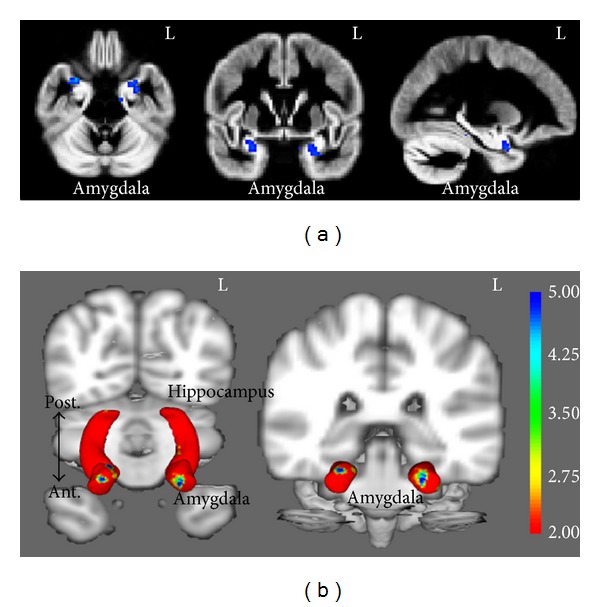
(a) shows whole-brain VBM analyses indicating significant group differences in the bilateral anterior amygdala (L = left). (b) shows SBM analyses of the amygdala and hippocampus indicating significant group differences in the anterior amygdala (post. = posterior, ant. = anterior). Color scale represents *t* values for the group comparison.

Whole brain VBM analyses controlling for overall GM volume revealed significant group differences in the bilateral anterior amygdala, such that the PTSD/mTBI group showed reduced volume, as compared to the control group (*P* < 0.05 TFCE corrected, 5000 permutations; see methods for a full description; Figure 1(a)). Because VBM analyses can be susceptible to incorrect registration and differences in individual cortical folding patterns [61], we also performed SBM on the amygdala to potentially corroborate our findings. SBM analyses mirrored our VBM analyses, indicating significant group differences in the bilateral anterior amygdala (*P* < 0.05 TFCE corrected, 5000 permutations; see methods for a full description; Figure 1(b)).

**Figure 2:**
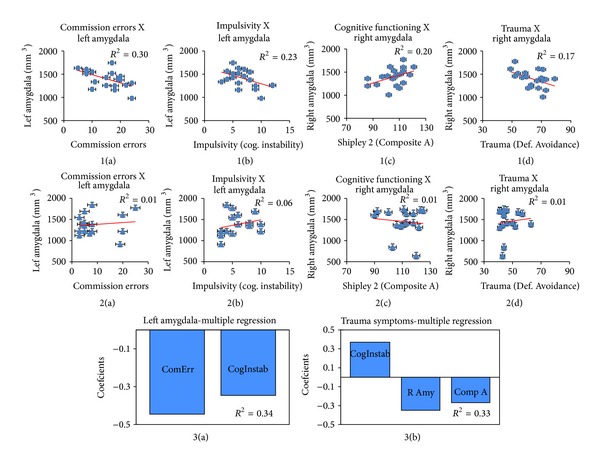
1(a)–1(d) show significant regression from the PTSD/mTBI group, while 2(a)–2(d) show the nonsignificant regression in the control group. 1(a)-2(a) show the relationship between commission errors and left amygdala volume (mm^3^); 1(b)-2(b) show relationship between impulsivity (subscale of cognitive instability, Barratt Impulsivity Scale) and left amygdala volume (mm^3^); 1(c)-2(c) show the relationship between cognitive functioning (Shipley 2 Composite A score) and right amygdala volume (mm^3^); 1(d)-2(d) show the relationship between trauma (Trauma Symptom Inventory subscale of Defensive Avoidance) and right amygdala volume (mm^3^). 3(a) shows the standardized coefficients for the multiple regression with left amygdala volume (ComErr = commission errors; CogInstab = the cognitive instability subcomponent of the Barratt Impulsivity Scale). 3(b) shows the standardized coefficients for the multiple regression with trauma symptoms (CogInstab = the cognitive instability subcomponent of the Barratt Impulsivity Scale, R Amy = right amygdala volume mm^3^; Comp A = Shipley 2 Composite A score). The multiple regression with left amygdala volume and multiple regression with trauma symptoms were controlled for whole brain grey matter volume

Next, to determine whether the volumetric differences in the amygdala are related to behavioral performance, we extracted an individual's left and right amygdala volume based on the SBM analyses and regressed it with Go/No-go errors of commission and omission, as well as RT, controlling for overall GM volume. Analyses revealed that errors of commission significantly related to volume in the left amygdala, such that increased errors of commission were predicted by decreased volume of the left amygdala, in the PTSD/mTBI group, but not the control group (*F* ^1,20^ = 7.81, *P* = 0.01, *R* ^2^ = 0.30; Figures 2(1(a)) and 2(2(a))).

We then determined whether the volume of the left and right amygdala was associated with impulsivity (Barratt), premorbid functioning (Shipley 2), and symptoms related to PTSD (Trauma Symptom Inventory) or TBI (OSU TBI-ID form), controlling for overall GM volume. Regression analyses revealed that an increased cognitive instability subcomponent of the Barratt Impulsivity Scale was predicted by decreased volume of the left amygdala in the PTSD/mTBI group, but not the control group (*F* ^1,20^ = 5.41, *P* = 0.03, *R* ^2^ = 0.23; Figures 2(1(b)) and 2(2(b))). Decreases of cognitive function as measured by Composite A of the Shipley 2 was associated with decreased volume of the right hippocampus in the PTSD/mTBI group but not the control group (*F* ^1,20^ = 4.60, *P* = 0.04, *R* ^2^ = 0.20; Figures 2(1(c)) and 2(2(c))). Increased scores on the Defensive Avoidance subscale of the Trauma Symptom Inventory were associated with decreased right amygdala volume in the PTSD/TBI group, but not the control group (*F* ^1,20^ = 4.84, *P* = 0.04, *R* ^2^ = 0.17; Figures 2(1(d)) and 2(2(d))). No relationship between amygdalar volume and symptoms of mTBI was noted.

With regard to hippocampal volume, our VBM and SBM analyses revealed no significant differences. We also tested the difference between the regression parameter estimates of amygdala volume with the behavioral variables and hippocampal volume with the behavioral variables, respectively. The differences in parameter estimates for amygdala volume and behavioral variables were all significantly different than those for hippocampal volume and behavioral variables, indicating that our findings are specific to amygdala volume (cognitive instability: *Z* = 2.05, *P* = 0.04; Shipley A: *Z* = −2.23, *P* = 0.03; Defensive Avoidance: *Z* = 2.49, *P* = 0.01).

As a summary analysis, we ran feature selection and multiple regression with left amygdala volume and multiple regression with trauma symptoms examining the sets of variables within the PTSD/mTBI group that best predicted (1) left amygdala volume (as it was found to be associated with motor inhibition and impulsivity) and (2) trauma symptoms related to PTSD (as they were the symptoms related to amygdala volume). Feature selection and multiple regression examining decreases in left amygdala volume were best predicted by a model indicating significant contributions of independent variance from both (a) increases in commission errors from Go/No-go performance and (b) increases in the cognitive instability subcomponent of the Barratt Impulsivity Scale *F* ^2,19^ = 5.94, *P* = 0.02, *R* ^2^ = 0.34; standardized coefficients are presented in Figure 2(3(a)). Feature selection and multiple regression examining increases in trauma symptoms related to PTSD were best predicted by a model indicating significant contributions of independent variance from (a) increases in the cognitive instability subcomponent of the Barratt Impulsivity Scale, (b) decreases in right amygdala volume, and (c) decreases of cognitive function of in Composite A of the Shipley 2 *F* ^3,18^ = 4.17, *P* = 0.04, *R* ^2^ = 0.33; standardized coefficients are presented in Figure 2(3(b)).

## 4. Discussion

The current study is the first, to our knowledge, in its approach to examine both voxel- and surface-based morphometry in a comorbid diagnostic group of individuals with PTSD/mTBI. Furthermore, it is novel in demonstrating that inhibitory control, as assessed through the Go/No-go task, is linked to known abnormalities in brain morphometry in PTSD/mTBI, namely, decreased amygdala volume. Decreased amygdala volume also was associated with increased impulsivity (Barratt Impulsivity Scale), a known indicator of deficits in inhibitory control. Both increased errors of commission and impulsivity contributed independent variance predicting decreases in amygdala volume. Furthermore, decreased amygdala volume was related to increases in trauma symptoms related to PTSD, but not mTBI symptoms (Trauma Inventory Scale, OSU TBI-ID, resp.). And finally, decreased amygdala volume, increased impulsivity, and decreased cognitive functioning assessed by the Shipley 2 Composite A contributed independent variance predicting increases in trauma symptoms related to PTSD.

While neuroimaging literature suggests that both singular diagnostic PTSD and TBI are related to decreases in the vmPFC/sgACC and amygdalar/hippocampal complex volume, our results indicate that comorbid diagnosis of PTSD/mTBI shows the same decreases in amygdala volume. Our analysis using VBM indicating reductions in anterior amygdala volume was corroborated by our analysis using SBM. The combination of VBM and SBM is an important analysis step considering that voxel-based morphometric studies have been shown to be susceptible to misregistration and individual cortical folding differences [61]. Utilizing both approaches and obtaining convergent findings enable us to be more confident in our results.

Although we found no group differences in the vmPFC/sgACC and the hippocampus, this null effect can potentially be attributed to a myriad of different factors. First, reductions of vmPFC/sgACC volume have been linked to TBI [62], but it is unclear how the severity of a TBI affects brain damage. Because individuals with a moderate or severe TBI were excluded from the current study, we may have less power to detect abnormalities in the vmPFC/sgACC. Additionally, mTBI has been linked to increased vulnerability of developing PTSD symptoms [6, 17], such that it may not be the long-term effects of mTBI, but rather a predisposition to developing PTSD in our sample that was detected. Future research is needed to determine whether there is a pattern whereby certain brain regions are not affected at low levels of severity of TBI, while other regions are affected more uniformly across levels of severity and the nature of mTBI predisposing individuals to PTSD. Second, while reductions in hippocampal volume have been replicated in a large number of studies examining PTSD, numerous studies also fail to replicate this finding [63, 64]. In particular, studies have suggested that only certain subpopulations (dependent on trauma type) show reductions in hippocampal volume [65]. Lastly, variation exists in the control samples used across these studies. One of the largest differences affecting results is likely because of the inclusion of either combat deployed or noncombat deployed military personnel. Because our study used previously combat deployed military OEF/OIF Veterans, differences between the current groups may be harder to detect than between PTSD/TBI and a nonmilitary control group. Future research is needed to determine whether simply the experience of combat deployment affects brain morphometry. For instance, combat deployment in general may relate to increased stress and subsequently affect the hippocampus through stress induced glucocorticoid release [66].

The current study analyzed multiple measures related to symptoms of both PTSD and mTBI. We indicate that deficits in inhibitory control, highly related to PTSD and TBI, are associated with decreases in amygdala volume in the PTSD/mTBI but not the control group. This relationship was significant in the PTSD/mTBI but not control group as indicated through regressions with errors of commission from the Go/No-go task, which also indicated significant group differences. Increased impulsivity, which has also been shown to be related to deficits in inhibitory control and performance on the Go/No-go task, also showed an association with decreased amygdala volume in the PTSD/mTBI but not control group. The Shipley 2 was used as a proxy for IQ, and therefore our best premorbid indicator of cognitive functioning. However, group differences emerged in both Abstraction and Composite A subscales, indicating that the PTSD/mTBI group exhibited decreased cognitive functioning. An individual's score on the Composite A subscale was also related to amygdala volume, such that decreased cognitive function were related to decreases in amygdala volume, in the PTSD/mTBI but not control group. We acknowledge that there are multiple interpretations regarding the Shipley 2 in the context of the current study (a) that the Shipley 2 is sensitive to the premorbid cognitive functioning of the individual, or (b) that the Shipley 2 is actually measuring cognitive impairment sequelae of PTSD and/or mTBI, or (3) some combination of the previous two interpretations. The current results are consistent with the literature suggesting that premorbid decreases in IQ may predispose individuals to PTSD [67]; however, it is notable that abstraction can be considered an executive function, which is known to be affected by both PTSD and TBI. Continued research is needed to determine the contribution of premorbid deficits and sequelae of individuals with PTSD and/or TBI as it relates to cognitive and executive function. The culmination of the findings from these multiple measures suggests that deficient inhibitory control is related to decreases in amygdala volume and may be more related to trauma associated with PTSD, rather than mTBI symptoms. The location of VBM and more importantly SBM findings in the anterior amygdala provide further interpretation of deficient inhibitory control as it relates to PTSD/mTBI.

Often the amygdala is treated as a functionally homogeneous region, but both animal and human research investigating nonpsychiatric populations suggests there may be dissociations of function along either a ventral-dorsal or anterior-posterior axis [68]. The ventral-dorsal axis has been linked to numerous functional dissociations including sensory input versus sensory output [69], impulsivity versus aggression [68], and fear conditioning (via input to the hippocampus) versus fear response [70], respectively. These dissociations are most likely attributed to the locality of two of the major amygdalar nuclei groups, the basolateral nuclei (BLA) and the central nuclei (CE). In humans, the BLA corresponds to the anterior or ventral segment of amygdala, while the CE is localized to the posterior or dorsal region of the amygdala [71]. The BLA is the set of nuclei in which multimodal sensory information converges [69]. This information is then processed and relayed to the CE nuclei group responsible for affecting physiological response via output to the brainstem, insula, and somatosensory cortex [70]. This well-established circuitry has been linked to fear extinction in which inhibitory cell groups (intercalated cell masses) positioned between the BLA and CE can reduce fear response via excitatory input from the vmPFC, which in turn decreases information flow to the CE nuclei group and fear mediated physiological response is lessened or abolished [69]. Therefore, our findings, indicating decreases specific to anterior amygdala volume, can likely be interpreted as abnormal morphometry associated with the BLA and as such may be linked to problems with multimodal sensory input. Problems with sensory input may lead to overprocessing of irrelevant stimuli and lead to increased anxiety and impulsive behavior [72–74]. Indeed, our results are consistent with this previous interpretation as we found that Go/No-go task, impulsivity, and trauma symptoms were linked to the anterior amygdala. Of course, it is not clear how reduced volume in this region is associated with sensory input specifically; however, it provides an interesting focus for future research.

The current study employed a standard cognitive task (Go/No-go), as opposed to using a symptom provocation task (e.g., script-driven imagery, processing of threat-related stimuli). Understandably, while most neuroimaging research regarding PTSD uses symptom provocation studies, it is also important to determine whether deficits in inhibitory function can be found under nonemotional conditions. The results of the current study suggest more general inhibitory control deficits in PTSD/mTBI individuals, consistent with the findings of Falconer et al., [47] in PTSD individuals. Therefore, future research using standard cognitive tasks may help to determine whether the neural systems affecting these populations are “generally” dysfunctional or whether they exhibit deficits only under certain conditions (e.g., threat/fear stimuli). Such information will likely be helpful to provide additional insights into how to develop more effective interventions.

While the current study uses multiple neuroimaging analysis techniques (i.e., VBM, SBM) and linear regression to indicate relationships between PTSD/mTBI symptoms and impairments, there are limitations that should be acknowledged. First, the sample size is relatively small for individual difference analyses, even though we first selected regions that exhibited group differences. Therefore, our findings are in need of replication. Second, we only included individuals with a mild but not moderate or severe TBI, which may have reduced our power to detect differences based on mTBI symptoms. Currently, it is unclear how TBI symptoms relate to differences in volume of specific brain regions. Third, our control population had trauma symptoms which have been shown to be linked to anatomical changes [75]; therefore, future studies need to determine the effect of trauma in isolation from diagnostic disorders. Fourth, our findings regarding increased impulsivity and the relation to decreased amygdala volume are based on the Barratt Impulsivity Scale, a self-report measure, which can lead to social desirability biases. Therefore, future research is needed to also investigate the relationship between impulsivity and amygdala volume with more biological indices of impulsivity. And last, we used a comorbid diagnostic group (i.e., PTSD/mTBI) to determine the relationships between brain morphometry and behavior/symptoms. Although this was the goal of the current study, future studies should examine the differences within a comorbid diagnostic group, in comparison to singular diagnostic groups (i.e., PTSD only, TBI only) to help fully decipher brain morphometry and the relationship to specific symptom profiles.

In sum, the current study has provided novel findings indicating abnormal anterior amygdala morphometry in comorbid PTSD/TBI, as compared to controls, as assessed by both voxel- and surface-based morphometric analyses. The group level reduction in amygdala volume was then shown to predict individual differences within the PTSD/mTBI group in errors of commission, impulsivity, cognitive function, and symptoms related to PTSD but not mTBI. Therefore, abnormalities in the anterior amygdala may provide a specific neural region that can be examined as it relates to deficits in inhibitory control, which may help identify biomarkers related more to PTSD, rather than mTBI in comorbid diagnostic groups.

## Figures and Tables

**Table 1:** Demographic and self-report information for the PTSD/TBI and control group. Numbers represent % *(N)* or median (range) in self-report measures. Fisher's exact or Wilcoxon rank sum test were used for all *P* values.

Characteristic	Control (*n* = 16)	TBI/PTSD (*n* = 21)	*P* value
Gender			0.57
Male	87.5% (14)	95.2% (20)	
Female	12.5% (2)	4.8% (1)	
Age	28.0 (24–45)	29.0 (23–43)	0.62
Race			0.37
Caucasian	75.0% (12)	90.5% (19)	
Other	25.0% (4)	9.5% (2)	
Years of education	15.5 (12–18)	14.5 (11–22)	0.14
Alcohol use			0.005
No history	81.3% (13)	38.1% (8)	
Past history of abuse	12.5% (2)	9.5% (2)	
Past history of dependence	6.3% (1)	52.4% (11)	
Substance use			0.60
No history	87.5% (14)	71.4% (15)	
Past history of abuse	6.3% (1)	9.5% (2)	
Past history of dependence	6.3% (1)	19.1% (4)	
Most severe TBI from deployment of related TBI			
Alteration of consciousness		38.1% (8)	
Loss of conscious < 5 minutes		52.4% (11)	
Loss of conscious 5 minutes to 30 minutes		9.5% (2)	
Number of symptoms from most recent injury		5 (0–9)	
Barratt Impulsivity Scale			
Attention	8.5 (5–15)	14 (9–19)	0.0002
Cognitive instability	5 (3–10)	6 (3–12)	0.16
Motor	14 (11–22)	15 (12–25)	0.11
Perseverance	7.5 (4–11)	9 (6–14)	0.01
Self-control	9 (6–16)	14 (6–22)	0.001
Cognitive complexity	10 (6–17)	12 (6–20)	0.09
Trauma Symptom Inventory			
Intrusive Experiences (IE)	45 (42–61)	75 (58–87)	<0.0001
Defensive Avoidance (DA)	44 (41–63)	67 (49–79)	<0.0001
Dissociation (DIS)	47 (41–55)	64 (47–98)	<0.0001
Shipley 2 Institute of Living Scale			
Vocabulary score	112 (99–121)	108 (86–121)	0.18
Abstraction score	108 (84–122)	93 (59–122)	0.004
Composite A score	112.5 (90–125)	99 (79–118)	0.006
